# lncRNA Mirt2 upregulates miR‐1246 through methylation to suppress LPS‐induced lung cell apoptosis

**DOI:** 10.1002/iid3.422

**Published:** 2021-05-04

**Authors:** Xuwen Xu, Yuyuan Xu, Xin Tao, Guangyu Liang

**Affiliations:** ^1^ Department of Infectious Diseases, Nanfang Hospital Southern Medical University Guangzhou China

**Keywords:** acute lung injury, lncRNA Mirt2, sepsis, miR‐1246, methylation

## Abstract

**Introduction:**

Long noncoding RNA Mirt2 has been proven to be a suppressor of lipopolysaccharide (LPS) (a key player in sepsis)‐induced inflammation responses. Therefore, Mirt2 may also participate in sepsis. This study was carried out to analyze the interactions between Mirt2 and microRNA‐1246 (miR‐1246) in sepsis, with a specific focus on sepsis‐induced acute lung injury (sepsis‐ALI).

**Methods:**

Forty sepsis patients (sepsis group; 23 males and 17 females; 40–65 years, 48.6 ± 6.3 years), 40 sepsis patients with acute lung injury (sepsis‐ALI group, 23 males and 17 females; 40–65 years, 48.7 ± 6.4 years), and 40 healthy controls (control group, 23 males and 17 females; 40–65 years, 48.6 ± 6.1 years) were included. Mirt2 and miR‐1246 expression in plasma samples from these patients were determined by a reverse transcription‐quantitative polymerase chain reaction (PCR). Overexpression of Mirt2 and miR‐1246 was achieved in human bronchial epithelial cells (HBEpCs) to explore the interaction between them. The effects of Mirt2 overexpression on miR‐1246 methylation were analyzed by methylation‐specific PCR. Cell apoptosis analysis was performed to analyze the role of Mirt2 and miR‐1246 in the apoptosis of HBEpCs.

**Results:**

Mirt2 expression was downregulated in sepsis and was further downregulated in patients with sepsis‐ALI. Mirt2 and miR‐1246 found to be positively correlated. Downregulation of Mirt2 and miR‐1246 was observed in HBEpCs with LPS treatment. In HBEpCs, Mirt2 overexpression increased miR‐1246 expression but decreased its gene methylation. Cell apoptosis analysis showed that Mirt2 and miR‐1246 negatively regulated the apoptosis of HBEpCs induced by LPS. In addition, miR‐1246 inhibition reduced the inhibitory effects of Mirt2 overexpression on cell apoptosis.

**Conclusions:**

Mirt2 may upregulate miR‐1246 through methylation to suppress lung cell apoptosis.

## Graphical abstract

Mirt2 and miR‐1246 are downregulated in sepsis and sepsis‐acute lung injury. Mirt2 can upregulate miR‐1246 through methylation to suppress lung cell apoptosis induced by lipopolysaccharide

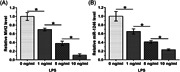

## 1 BACKGROUND

In certain cases, the body's systemic immune and inflammatory responses can be dysregulated in response to infections, leading to the development of sepsis.^1^, ^2^ Though most patients will recover from mild sepsis, more than 40% of patients with sepsis shock will die of this disease.^3^, ^4^ The high mortality rate of severe sepsis patients is closely linked with multiple organ dysfunctions, but the underlying mechanism of organ dysregulation and failure in sepsis patients remains elusive.^5^ Acute lung injury (ALI) is frequently observed in sepsis patients.^6^, ^7^ In spite of the efforts made on the development of novel treatment approaches, treatment outcomes of ALI in sepsis patients remain unsatisfactory.^6^, ^7^

MicroRNAs and long noncoding RNAs (lncRNAs) regulate gene expression and participate in human diseases.^8^, ^9^ Differentially expressed ncRNAs have also been identified in sepsis and certain ncRNAs have been proven to be critical in sepsis.^10^, ^11^, ^12^ However, the function of most ncRNAs in sepsis remains unclear. lncRNA Mirt2 is a recently characterized negative regulator of lipopolysaccharide (LPS)‐induced inflammation^13^ and plays critical roles in sepsis.^14^ Our preliminary RNA‐seq analysis revealed altered Mirt2 expression in sepsis and its positive correlation with microRNA‐1246 (miR‐1246), which can inhibit ALI inflammatory responses.^15^ Therefore, in this study, we analyzed the interaction between Mirt2 and miR‐1246 in sepsis, with a specific focus on sepsis‐induced ALI (sepsis‐ALI).

## 2 METHODS

### 2.1 Research subjects

The study included 40 sepsis patients (sepsis group, 23 males and 17 females, 40 to 65 years old with a mean of 48.6 ± 6.3), 40 sepsis‐ALI patients (sepsis‐ALI group, 23 males and 17 females, 40 to 65 years old with a mean of 48.7 ± 6.4) and 40 healthy controls (control group, 23 males and 17 females, 40 to 65 years old with a mean of 48.6 ± 6.1). All participants were enrolled at Nanfang Hospital, Southern Medical University between May 2017 and May 2019. All sepsis and sepsis‐ALI patients were caused by bacterial infections. Patients who had initiated therapy before admission and patients who were diagnosed with other severe clinical disorders were excluded.

### 2.2 Plasma samples and human bronchial epithelial cells (HBEpCs)

Fasting blood (3 ml) was extracted from each participant and plasma samples were prepared.

HBEpCs from Sigma‐Aldrich were cultured in Airway Epithelial Cell Growth Medium (PromoCell) following the manufacturer's instructions.

Murine alveolar macrophages cell line MH‐S purchased from the American Type Culture Collection (ATCC) were routinely passaged in RPMI1640 (Nacalai Tesque) supplemented with 10% heat‐inactivated fetal bovine serum and 100 U/ml penicillin–streptomycin at 37°C in a humidified incubator with 5% CO_2_.

### 2.3 Cell transfections

pcDNA3.1‐Mirt2 was constructed. miR‐1246 and its negative control (NC) miRNA, Mirt2 small interfering RNA (siRNA), and its NC siRNA, as well as miR‐1246 inhibitor and its NC, were from Sigma‐Aldrich. Cells transfected with pcDNA3.1‐Mirt2 or control pcDNA3.1 vectors (10 nM), Mirt2 (40 nM) or NC siRNAs, MiR‐1246 or NC miRNAs (40 nM), miRNA or NC inhibitors (40 nM), as well as untransfected control cells (C) were included in all experiments. Transfection was performed by incubating cells for 6 h with the mixtures prepared with the above reagents and Lipofectamine 2000 (Invitrogen) following the manufacturer's instruction. After that, cells were washed three times with a fresh medium and subsequent experiments were performed 48 h later.

### 2.4 RNA preparations

RNAzol (Sigma‐Aldrich) was used for RNA extraction. To explore the effects of LPS treatment on the expression of Mirt2 and miR‐1246, HBEpCs were incubated with a medium containing 0, 1, 5, or 10 ng/ml LPS (Cat # L2630‐10MG; Sigma‐Aldrich) for 48 h before RNA preparation. Genomic DNA was removed using DNase I. The standard curve was prepared by performing 10‐fold serial dilutions of the complementary DNA (cDNA) template, after which, quantitative polymerase chain reactions (qPCRs) were performed to determine their threshold cycle values. The linear fitting curve of the threshold cycle versus the dilution factor was plotted and the correlation coefficient (*R* ^2^) for the line was .99 or greater.

cDNA samples were prepared and used as templates for qPCR analyses with 18S ribosomal RNA as the internal control to measure Mirt2 expression. miR‐1246 expression was analyzed using All‐in‐One™ miRNA quantitative reverse transcription PCR (qRT‐PCR) reagent kit (GeneCopoeia) with U6 as the internal control. The method of $2^{‐\Delta \Delta C_{t}}$ was used for data normalization. PCR thermal cycling conditions were 10 min at 95°C followed by 40 cycles of 10 s at 95° and 55 s at 57°.

### 2.5 Methylation‐specific PCR (MSP)

A DNA Extraction Kit (Agilent) was used to extract genomic DNA from HBEpCs followed by conversion using DNA Methylation‐Gold™ kit (ZYMO Research). Taq DNA polymerase kit (NEB) was used to carry out MSP to measure the methylation level of the miR‐1246 gene.

### 2.6 Cell apoptosis analysis

HBEpCs were treated with 10 ng/ml LPS for 48 h. After washed with ice‐cold phosphate‐buffered saline, cells were stained with propidium iodide and fluorescein isothiocyanate‐annexin V and analyzed by flow cytometry to evaluate their apoptotic status. All steps were completed in strict accordance with the manufacturer's instructions.

### 2.7 Statistical analysis

Analysis of variance Tukey's test was used for all comparisons. Linear regression was used for association analysis. *p* < .05 was considered statistically significant.

## 3 RESULTS

### 3.1 Mirt2 and miR‐1246 were downregulated in sepsis and sepsis‐ALI patients

**Figure 1:**
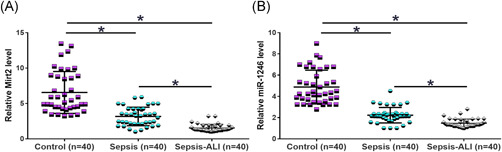
Mirt2 and miR‐1246 are downregulated in sepsis and sepsis‐ALI patients. Levels of Mirt2 (A) and miR‐1246 (B) in plasma samples from sepsis patients (*n* = 40), sepsis‐ALI patients (*n* = 40), and controls (*n* = 40) were measured by performing reverse transcription‐quantitative polymerase chain reaction. ALI, acute lung injury; miR, microRNA. *p < .05

Mirt2 and miR‐1246 expression levels in sepsis patients (*n* = 40), sepsis‐ALI patients (*n* = 40), and controls (*n* = 40) were determined by RT‐qPCR. Mirt2 (Figure 1A) and miR‐1246 (Figure 1B) were found to be downregulated in sepsis patients and even further downregulated in sepsis‐ALI patients (*p* < .05).

### 3.2 Mirt2 and miR‐1246 were positively correlated

**Figure 2:**
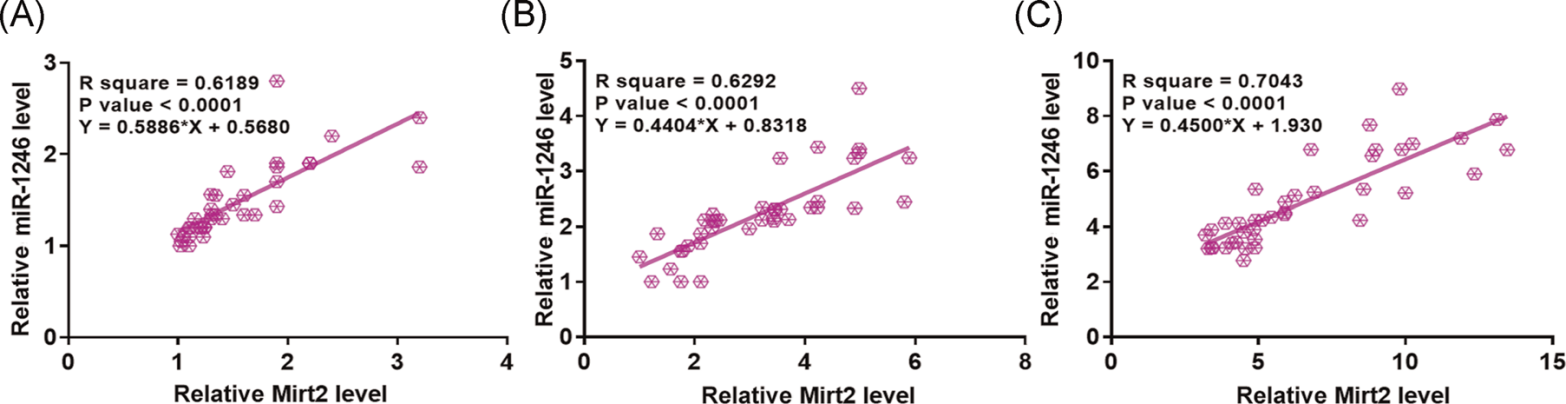
Mirt2 and miR‐1246 are positively correlated. Linear regression was performed to analyze the correlation between Mirt2 and miR‐1246 across plasma samples from sepsis‐ALI patients (A), sepsis patients (B), and controls (C). ALI, acute lung injury; miR, microRNA

Linear regression was performed to analyze the correlation between Mirt2 and miR‐1246 across plasma samples from sepsis‐ALI patients (*n* = 40), sepsis patients (*n* = 40), and controls (*n* = 40). Mirt2 and miR‐1246 were found to be positively correlated across sepsis‐ALI (Figure 2A), sepsis samples (Figure 2B), and control samples (Figure 2C).

### 3.3 LPS treatment downregulated Mirt2 and miR‐1246 in HBEpCs

**Figure 3:**
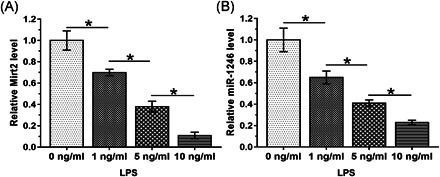
LPS treatment downregulates Mirt2 and miR‐1246 in HBEpCs. To explore the effects of LPS treatment on the expression of Mirt2 (A) and miR‐1246 (B), HBEpCs were incubated with medium containing 0, 1, 5, or 10 ng/ml LPS for 48 h, followed by the measurement of the expression levels of Mirt2 and miR‐1246 by performing reverse transcription‐quantitative polymerase chain reaction. HBEpC, human bronchial epithelial cell; LPS, lipopolysaccharide; miR, microRNA. *p < .05

To explore the effects of LPS treatment on expression of Mirt2 and miR‐1246, HBEpCs were incubated with a medium containing 0, 1, 5, or 10 ng/ml LPS for 48 h, followed by RT‐qPCR analyses to measure expression levels of Mirt2 and miR‐1246. LPS treatment was found to decrease Mirt2 (Figure 3A) and miR‐1246 (Figure 3B) expression levels in a dose‐dependent manner.

### 3.4 Mirt2 overexpression leads to increased miR‐1246 expression but decreased miR‐1246 gene methylation

**Figure 4:**
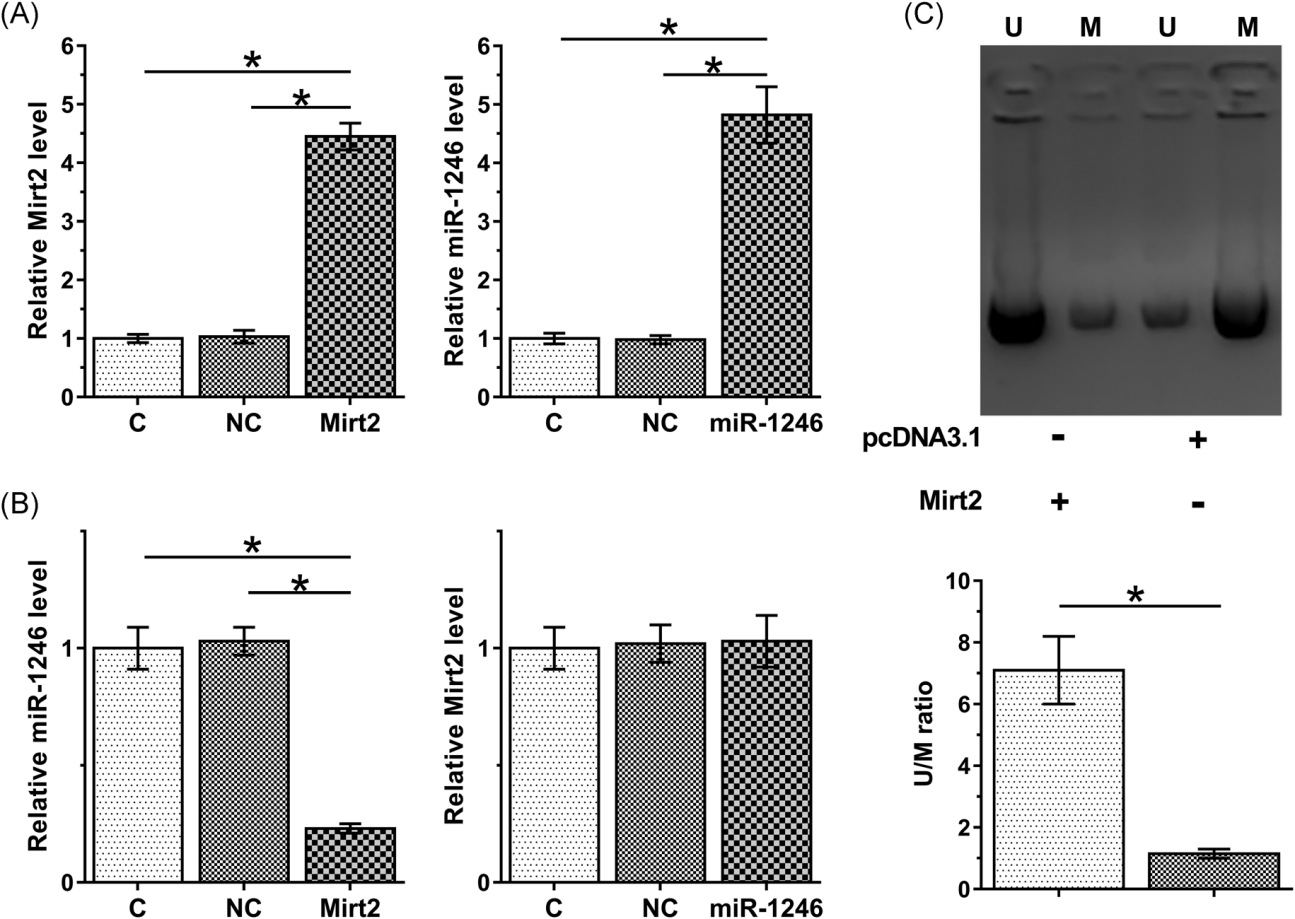
Mirt2 overexpression leads to the upregulation of miR‐1246 and decreased methylation of the miR‐1246 gene. Mirt2 and miR‐1246 were overexpressed (A). The effects of Mirt2 and miR‐1246 on each other (B) were also analyzed by a reverse transcription‐quantitative polymerase chain reaction. The methylation‐specific polymerase chain reaction was performed to analyze the effects of Mirt2 overexpression on the methylation of the miR‐1246 gene (C). M, methylation; miR, microRNA; U, unmethylation. *p < .05

Both Mirt2 and miR‐1246 were overexpressed (Figure 4A, *p* < .05). Mirt2 overexpression increased miR‐1246 level (*p* < .05), whereas miR‐1246 overexpression showed no significant effect on Mirt2 (Figure 4B). MSP data illustrated that Mirt2 expression vector transfection significantly increased the level of the methylated‐PCR product (Figure 4C).

### 3.5 MiR‐1246 inhibition alleviated the inhibitory effect of Mirt2 on LPS‐induced inflammatory response in murine alveolar macrophages

**Figure 5:**
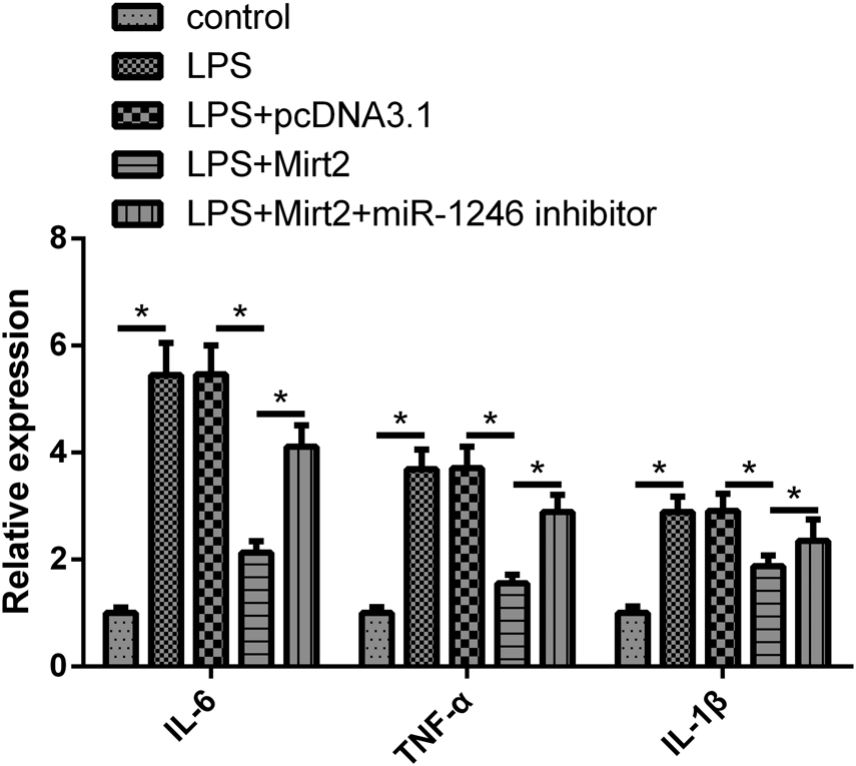
miR‐1246 inhibition alleviates the inhibitory effect of Mirt2 on LPS‐induced inflammatory response in murine alveolar macrophages. The mRNA expression levels of IL‐6, TNF‐α, and IL‐1β in MH‐S cells transfected with pcDNA3.1, pcDNA3.1‐Mirt2, and pcDNA3.1‐Mirt2 + miR‐1246 inhibitor were estimated by a reverse transcription‐quantitative polymerase chain reaction. IL, interleukin; LPS, lipopolysaccharide; miR, microRNA; mRNA, messenger RNA; TNF‐α; tumor necrosis factor‐α. *p < .05

To investigate whether Mirt2 regulates LPS‐induced inflammatory response via miR‐1246, messenger RNA (mRNA) levels of inflammatory cytokines interleukin‐6 (IL‐6), tumor necrosis factor‐α (TNF‐α), and IL‐1β were measured in MH‐S cells transfected with pcDNA3.1, pcDNA3.1‐Mirt2, and pcDNA3.1‐Mirt2 + miR‐1246 inhibitors, respectively. The results showed that Mirt2 significantly suppressed LPS‐induced production of IL‐6, TNF‐α, and IL‐1β in MH‐S cells, and these suppressions were partially attenuated by miR‐1246 inhibition (Figure 5).

### 3.6 Mirt2 and miR‐1246 suppressed LPS‐induced HBEpCs apoptosis

**Figure 6:**
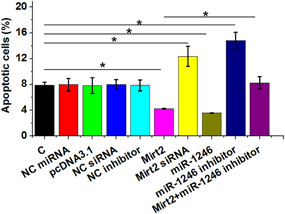
Mirt2 and miR‐1246 suppress the apoptosis of HBEpCs induced by LPS. Cell apoptosis assay was performed to analyze the effects of the overexpression and inhibition of Mirt2 and miR‐1246 on the apoptosis of HBEpCs induced by LPS. HBEpC, human bronchial epithelial cell; LPS, lipopolysaccharide; miR, microRNA; NC, negative control; siRNA, small interfering RNA. *p < .05

Cell apoptosis assay was performed to analyze the role of Mirt2 and miR‐1246 in regulating the apoptosis of HBEpCs induced by LPS. It is worth noting that 10 ng/ml LPS was chosen for its significantly stronger effect on Mirt2 and miR‐1246 expression than LPS at lower concentrations and its significantly stronger on gene expression than LPS at higher concentrations. It was observed that Mirt2 and miR‐1246 overexpression decreased the apoptotic rate of HBEpCs induced by LPS, whereas Mirt2 siRNA silencing and miR‐1246 inhibitor treatment increased cell apoptosis. In addition, cotransfection of Mirt2 and miR‐1246 inhibitor showed that miR‐1246 inhibitor treatment reversed the inhibitory effect of Mirt2 overexpression on cell apoptosis (Figure 6, *p* < .05).

## 4 DISCUSSION

This study mainly investigated the interactions between Mirt2 and miR‐1246 in sepsis‐ALI. We found that Mirt2 and miR‐1246 were downregulated in both sepsis and sepsis‐ALI patients and that Mirt2 may upregulate miR‐1246 through methylation to suppress LPS‐induced lung cell apoptosis.

Mirt2 has been proven to be a negative regulator of multiple inflammatory diseases such as ulcerative colitis, in which Mirt2 is downregulated and regulates the expression of IL‐22 to suppress cell apoptosis.^16^ In addition, it is also proven to inhibit LPS‐induced PC12 cell injury by downregulating the expression of miR‐429.^17^ Sepsis is also a type of inflammatory disease, in which LPS plays a critical role. In fact, LPS‐treated cells are widely used as the cell model of sepsis.^14^ This study is the first to report downregulation of Mirt2 in sepsis and further downregulation in sepsis patients with ALI. In addition, overexpression of Mirt2 results in a reduced apoptotic rate of lung cells under LPS treatment. Therefore, Mirt2 may play a protective role in lung cells from patients with sepsis.

Interestingly, a recent study reported that LPS treatment increases Mirt2 expression in macrophages through interactions with p38‐Stat1 and interferon‐Stat1 pathways, and identifies lncRNA Mirt2 as a negative feedback regulator of excessive inflammation.^13^ Moreover, Bai et al.^18^ indicated that LPS suppresses Mirt2 expression and overexpression of Mirt2 mitigates LPS‐induced inflammatory damages in HK‐2 cells. Similarly, Li et al.^19^ found that lncRNA Mirt2 level is upregulated in LPS‐stimulated PC12 cells and serum samples derived from spinal cord injury patients, and overexpression of lncRNA Mirt2 protects PC12 cells from LPS‐induced injuries. Our study also showed that LPS downregulates Mirt2 in lung cells. Therefore, LPS may play an opposite role in regulating Mirt2 expression in different types of cells.

In a recent study, Suo et al.^15^ reported that miR‐1246 suppresses ALI‐induced inflammatory responses and lung cell apoptosis by interacting with Wnt/β‐catenin and nuclear factor‐κB pathways. Similarly, our study also showed the inhibitory effects of miR‐1246 on the apoptosis of lung cells induced by LPS. Moreover, Mirt2 overexpression significantly inhibits LPS‐induced production of IL‐6, TNF‐α, and IL‐1β in MH‐S cells, and these effects are partially neutralized by miR‐1246 inhibition. lncRNAs are well known for regulating the expression of miRNAs through methylation.^20^ Consistently, in the present study, we found that Mirt2 upregulates miR‐1246 by decreasing miR‐1246 gene methylation. However, the methylation‐related factors that mediate this process remain unknown. Further in‐depth investigations are needed.

## 5 CONCLUSIONS

In conclusion, Mirt2 and miR‐1246 are downregulated in sepsis and sepsis‐ALI and Mirt2 upregulates miR‐1246 through methylation to suppress lung cell apoptosis induced by LPS.

## CONFLICT OF INTERESTS

The authors declare that there are no conflict of interests.

## AUTHORS' CONTRIBUTIONS

Xuwen Xu conducted literature research, designed and carried out the experiments, analyzed the data, and wrote the manuscript; Yuyuan Xu, Guangyu Liang, and Xin Tao conducted literature research, carried out the experiments, and performed statistical analyses. All authors have read and approved the final manuscript.

## ETHICS APPROVAL AND CONSENT TO PARTICIPATE

This study was approved by the Ethics Committee of Nanfang Hospital, Southern Medical University. World Medical Association Declaration of Helsinki was followed. All participants had provided written informed consent.

## Data Availability

The datasets used and analyzed during the current study are available from the corresponding author on reasonable request.
